# The Expression Levels of Toll-like Receptors after Metallic Particle and Ion Exposition in the Synovium of a Murine Model

**DOI:** 10.3390/jcm10163489

**Published:** 2021-08-07

**Authors:** Xiangyun Cheng, Volkmar Jansson, Jan Philippe Kretzer, Rainer Bader, Sandra Utzschneider, Alexander C. Paulus

**Affiliations:** 1 Department of Orthopedic Surgery, Physical Medicine and Rehabilitation, University Hospital of Munich, Ludwig-Maximilians-University, Campus Großhadern, Marchioninistraße 15, 81377 Munich, Germany; volkmar.jansson@med.uni-muenchen.de (V.J.); prof@ortho-utzschneider.de (S.U.); Alexander.Paulus@med.uni-muenchen.de (A.C.P.); 2 Laboratory of Biomechanics and Implant Research, Clinic for Orthopedics and Trauma Surgery, Heidelberg University Hospital, Schlierbacher Landstraße 200a, 69118 Heidelberg, Germany; philippe.kretzer@med.uni-heidelberg.de; 3 Biomechanics and Implant Technology Research Laboratory (FORBIOMIT), Department of Orthopaedics, Rostock University Medical Center, Doberaner Straße 142, 18057 Rostock, Germany; rainer.bader@med.uni-rostock.de

**Keywords:** TLR, metallic particles, metallic ions, inflammatory response, corrosion, synovium

## Abstract

To date, the exact role of specific Toll-like receptors (TLRs) in regulating immune reactivity to metallic byproducts of orthopedic implants has not been fully clarified. In light of the situation, our objective in this investigation was to assess the expression levels of surface TLRs after metallic particle and ion exposure in an established animal model. Ten female BALB/c mice in each group received intra-articular injections of phosphate buffer (PBS) (control), metallic particles (MP), and metallic ions (MI), respectively. Seven days later, immunohistochemical staining was undertaken in the synovial layer of the murine knee joints using anti-TLR 1, 2, 4, 5, and 6 polyclonal antibodies. In addition to increased cellular infiltrates and a hyperplastic synovial membrane, the MP group showed significantly elevated TLR expression compared to the control group and had higher TLR 1-, 4-, and 6-positive cells than the MI group (*p* < 0.0167). TLR 4- and TLR 6-positive cells were significantly augmented for the MI group compared to the control group (*p* < 0.0167). Additionally, greenish corrosion particles found in the necrotic tissue suggested that metallic particles might release a certain level of locally toxic metallic ions in vivo.

## 1. Introduction

Due to electrochemical corrosion and mechanical wear, almost all metals and metallic alloys used for artificial joints will inevitably release metallic ions and wear particles in the human body, which causes periprosthetic biological reactions followed by aseptic implant loosening [1,2]. Despite advances in surgical techniques and implant design, aseptic implant loosening related to adverse biological reactions remains one common long-term complication of joint arthroplasty. It might ultimately result in revision surgery, which has higher technical difficulty, cost and complication rates than primary surgery [3,4]. To fully elucidate the complicated mechanism of metal wear or corrosion-associated biological reactions is imperative, not only for diagnosis and prevention but also for further therapies of aseptic implant loosening.

In clinically histopathological studies, periprosthetic tissues from revision surgery, especially peri-implant tissue of metal-on-metal (MoM) bearing couples, commonly show a synovial-like membrane, in which wear particles, macrophages, giant cells, lymphocytes, dendritic cells, fibroblasts, and endothelial cells infiltrate [5,6]. Occasionally, granuloma formation is observed around the prosthesis [7]. Furthermore, upregulated expression of pro-inflammatory cytokines, such as tumor necrosis factor-α (TNF-α), interleukin-1β (IL-1β), and interleukin-6 (IL-6) are frequently detected in retrieved tissues associated with aseptic implant loosening [8,9]. Thus, based on this clinical evidence, it has been commonly accepted that wear particles and high levels of metallic ions cause sterile inflammatory reactions characterized by the continuous recruitment of immunocompetent cells, release of pro-inflammatory mediators, and sometimes formation of granulomatous tissues (pseudotumors) [10]. These adverse events will result in peri-implant osteolysis, followed by aseptic loosening in the mid/long term [2]. Whilst the general mechanism of sterile inflammation caused by wear particles and metallic ions has been established as described above, some detailed issues, such as the bioactive difference caused by between metallic particles and ions and exact patterns of the ligand-receptor recognition related to metallic particles and ions, remain controversial [11,12,13].

Toll-like receptors (TLRs), as one type of pattern recognition receptors (PRRs), can not only recognize pathogen- and danger-associated molecular patterns (PAMPs and DAMPs), but also transduce signals closely associated with the induction of pro-inflammatory mediators (IL-6, TNF-α, and IL-1β). Thus, they are likely to play a critical role in recognizing metallic particles and ions and initiating peri-implant sterile inflammation [14,15,16]. Especially for some specific TLRs found in both humans and mice (i.e., TLRs 1, 2, 4, 5, and 6), these receptors are usually expressed on the surface of various immune cells, increasing the possibility of direct interactions with wear debris [17,18,19]. Furthermore, previous studies have shown that, after the exposure of various endotoxin-free particles (e.g., polymers [20], hydroxyapatite [21], and titanium [22]), distinct TLR expression of different immune cells were elevated in vitro and in vivo, indicating the activation of immune cells related to inflammatory reactions [23,24,25]. However, each known TLR can bind many different ligands, and is even referred to as the promiscuous receptor [26,27]. For instance, TLR 4 can recognize lipopolysaccharide (LPS) and some endogenous ligands (Heat shock protein 60, α-synuclein, and fibrinogen) [28]. Due to the functional complexity of TLRs, the exact role of each TLR in mediating immune reactivity to specific wear debris is still not fully understand [11].

Cobalt-chromium-molybdenum (Co28Cr6Mo) alloys represent the preferred material for MoM hip endoprostheses. Nevertheless, in the late 2000s, due to the issues of metal particles and ions, the MoM replacements were almost stopped completely [29]. However, nearly a million postoperative patients still used the MoM implants after issues of “debris disease” came to light. Therefore, to fully understand the adverse reactions caused by metal particles and ions is critical. Numerous studies for Co28Cr6Mo implants have been conducted in vivo and in vitro and indicated that Co28Cr6Mo nanoparticles were cytotoxic and prompted cell apoptosis, and at higher dose, necrosis, with inflammatory reactions [30]. Furthermore, utilizing Co28Cr6Mo particles and ions, researchers once demonstrated that Co28Cr6Mo particles could induce more intense sterile inflammatory responses than Co28Cr6Mo ions in the synovial membrane in vivo [31]. According to some studies, wear debris-induced inflammation might be mitigated by specific pharmacological blockade of one or more of these TLRs [11,32]. However, to our knowledge, in terms of the relationship between TLRs and Co28Cr6Mo implant byproducts, especially Co28Cr6Mo ions, related studies in vivo were scarce, not to mention the comparative study of TLR activation between Co28Cr6Mo particles and Co28Cr6Mo ions in the synovial membrane.

In light of the current situation, standard Co28Cr6Mo alloys were chosen in this investigation, and the objective was to determine the potential changes in cell surface receptors (specific TLRs) in response to metallic particles (MP) and metallic ions (MI), and to distinguish the difference in TLR activation between metallic particles and metallic ions in the synovial membrane of murine knee joints. The hypothesis was that the group injected with metal particles (MP) and the metal ion group (MI) would express cell surface TLRs at higher levels. Because metal ions can function as haptens, the MI group would exhibit higher expression of specific TLRs than the MP group.

## 2. Materials and Methods

### 2.1. Co28Cr6Mo Particles/Ions Generation

Specimens of a Co28Cr6Mo alloy following ISO 5832–12/ASTM F1537 (standard alloys in hip and knee arthroplasties) [33] were used to produce metal particles and metal ions.

In order to obtain metal wear particles, a custom-made pin-on-plate simulator was utilized to run a wear test at a frequency of 1 Hz. The generated particle chemical composition was examined by a high-resolution inductively coupled plasma mass spectrometry (HR-ICP-MS) instrument (Thermo Scientific, Bremen, Germany). Additionally, the aspect ratio (AR), equivalent circular diameter (ECD), and the roundness (R) of wear particles were determined utilizing scanning electron microscopy (SEM; Carl Zeiss, Oberkochen, Germany). According to our previous results, these generated particles have a mean size in the nanometer range (ECD: 61.25 ± 18.47 nm) with an aspect ratio of 1.69 ± 0.66 and a roundness of 0.64 ± 0.16 [34]. Additionally, the shape of metal particles was principally round and oval, accompanied by a small proportion of needle-shaped particles, similar to wear particles in retrieved tissues of revision surgeries [35,36].

Solid Co28Cr6Mo samples were immersed in a PBS solution and served as an anode against a hydrogen bridge electrode in a corrosion measuring cell to generate metal ions. After being analyzed by an HR-ICP-MS instrument (Thermo Scientific, Bremen, Germany), the content of all dissolved metal ions in the stock solution was determined as 20.5 mg/L, which was subsequently diluted to the target concentration of 200 μg/L utilizing the PBS solution (Table 1). The selected concentration of 200 μg/L was based on analytical research of patients’ joint aspiration before revision surgeries, which showed these metal ions’ median concentrations (mainly in the range of 200–250 μg/L) [37].

### 2.2. Removal of Endotoxins

The obtained metallic wear particles and ions would be used to induce TLR activation in the synovial membrane in vivo. Before the formal experiment, PAMPs, especially lipopolysaccharides (LPS; TLR 4 ligand), had to be eliminated to avoid relevant disturbances. Therefore, the obtained particles were cleaned by an ethanol washing procedure, whereas metallic ion solutions were heat shocked. The removal of endotoxins was examined by the Limulus amebocyte lysate (LAL) assay (Lonza, Cologne, Germany).

### 2.3. Animals and Intraarticular Injection

Thirty female BALB/c mice (Charles River Wiga, Sulzbach, Germany) with a mean age of seven weeks, weighing 18–25 g, were housed and fed in the Walter Brendel Centre of Experimental Medicine at Ludwig Maximilian University of Munich. All animals were randomly divided into three groups: the MP group (n = 10), the MI group (n = 10), and the PBS control group (n = 10).

Before the intra-articular injection process, solutions of all groups were sonicated for 60 min to avoid potential precipitation and aggregation. Subsequently, 50 μL of a 0.1 vol% metallic particle (MP) suspension, 50 μL of 200 μg/L metallic ions (MI) solution, and 50 μL of the PBS solution were injected into the murine left knees under sterile conditions. After seven days, the mice were euthanized by an overdose of pentobarbital (Merial GmbH, Hallbergmoos, Germany), followed by an acquisition of all knee joints for the subsequent immunohistological analysis.

### 2.4. Immunohistochemistry

All knee joints were fixed in 4% formaldehyde (Microcos GmbH, Garching, Germany), followed by the decalcification using Osteosoft^®^ solution (Merck, Darmstadt, Germany) at room temperature. Decalcified samples were then dehydrated in a Spin Tissue Processor-120 (Myr, Tarragona, Spain) and processed for paraffin wax embedding. For performing the subsequent staining procedures, paraffin wax sections were cut at 3 μm thickness and mounted on Superfrost Plus glass slides (Menzel, Braunschweig, Germany).

In order to avoid the false-positive staining and the false-negative staining, no primary antibody controls (NC) and positive controls were established by using knee samples (without adding primary antibodies) and splenic samples (rich in TLRs), respectively. For the heat-induced epitope retrieval (HIER) procedure, EDTA buffer pH 8 (DCS, Hamburg, Germany) was selected based on a pre-test. The optimized dilution factor for each polyclonal antibody was got by serial dilution pre-tests: TLR 1 (1: 500 dilution), TLR 2 (1: 200 dilution), TLR 4 (1: 200 dilution), TLR 5 (1: 300 dilution) and TLR 6 (1: 200 dilution) (Biorbyt Ltd., Cambridge, UK). After these pre-tests, the formal staining procedures were subsequently performed. Briefly, when the endogenous peroxidase blocking procedure (3% H_2_O_2_, Merck, Darmstadt, Germany) was finished, tissue sections were incubated with primary antibodies for one hour. After the SuperVision 2 HRP-Polymer system kit (DCS, Hamburg, Germany) was used to bind primary antibodies, tissue sections were incubated with the DAB-Kit (DCS, Hamburg, Germany) for 3 min and then counterstained with Mayer’s hematoxylin (Morphisto GmbH, Frankfurt, Germany), and mounted using cover-slips. Before every step during the staining period, tissue sections were washed in the washing buffer (PBS-Brij solution, 1000:1, Sigma-Aldrich, Germany) three times.

Immunohistochemically stained sections were examined with a M8 microscope (PreciPoint, Freising, Germany) at 200× magnification. Images containing most of the synovial tissue (region of interest, ROI) were captured for following histomorphometrical analysis in the present study. If the cell in the synovium was stained light yellow or brown, and the nucleus was stained blue, positive staining was recorded. Manual counts of the collected images were conducted individually by two participants utilizing Image J software (National Institute of Mental Health, Bethesda, MD, USA). The average value would be used to perform the following statistical analysis.

### 2.5. Ethics

All experimental steps involving animals in this project were approved by the government of Oberbayern, Bavaria, Germany (Protocol number: 55.2-1-54-2532-82.12). All steps were done following the rules and regulations of the Animal Protection Laboratory Animal Regulations (2013), European Directive 2010/63/EU and rules and regulations in Ludwig Maximillian University of Munich (LMU), Bavaria, Germany Tierschutzgesetz §1/§4/§17 (https://www.gesetze-im-internet.de/tierschg/TierSchG.pdf, accessed on: 20 July 2021).

### 2.6. Statistics

All data were analyzed with GraphPad Prism (Version 8.3.0, GraphPad Software, San Diego, CA, USA). Data were presented using box plots. The distribution of the data sets was tested by using the Anderson–Darling test and the Shapiro–Wilk test. Groups with normal distribution were analyzed using a one-way analysis of variance (ANOVA) followed by Tukey’s multiple comparisons test. If the data obtained failed the normality test, a nonparametric test (Kruskal–Wallis) followed by Dunn’s multiple comparisons test would be conducted. *p*-values were adjusted by the Bonferroni correction and considered to be statistically significant below 0.0167.

## 3. Results

### 3.1. Expression of TLR 1

**Figure 1:**
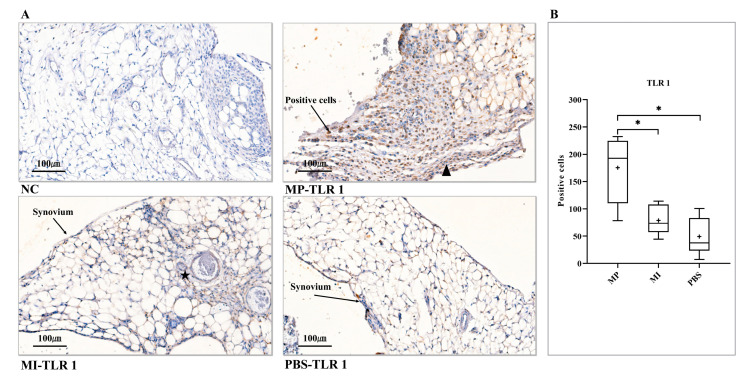
Expression of TLR 1 in the synovial layer of knee joints. (**A**) NC, no positive cell was found. MP-TLR 1, rounded macrophage-like cells (black arrows), spindle-shaped fibroblast-like cells (▲). MI-TLR 1, the capillary in the adjacent adipose tissue (★). PBS-TLR 1, scattered positive cells were found in the synovial membrane. (**B**) Histomorphometrical analysis of TLR 1 expression levels in three groups (One-way ANOVA (Tukey’s test)). (+) symbol represents the mean value. NC, no primary antibody control; MP, metal particles; MI, metal ions; PBS, phosphate-buffered saline; TLR, Toll-like receptor. (Scale bars = 100 µm; * = *p* < 0.0167).

The positive control staining (Figure S1) showed that the optimized staining procedure was working correctly; the NC staining (Figure 1A, NC) proved the staining specificity. Concerning the MP group (Figure 1A, MP-TLR 1), numerous rounded macrophage-like cells and intensive spindle-shaped, fibroblast-like cells were found in the central and peripheral regions of the hyperplastic synovial layer, respectively; most of them were positive for the TLR 1 antibodies. In a representative image of the MI group (Figure 1A, MI-TLR 1), the synovial tissue showed a tendency to “invade’’ the adjacent adipose tissue. Some TLR 1-positive cells around the capillaries of the adjacent adipose tissue were found. For the PBS group (control), there were only scatted positive TLR 1 cells in the synovial tissue. Histomorphometrical analysis indicated that the expression of TLR 1 in the MP group was considerably elevated compared with the MI group and the PBS group (*p* < 0.0167); however, no statistically apparent difference was found between the MI group and the PBS group (*p* = 0.262) (Figure 1B).

### 3.2. Expression of TLR 2

**Figure 2:**
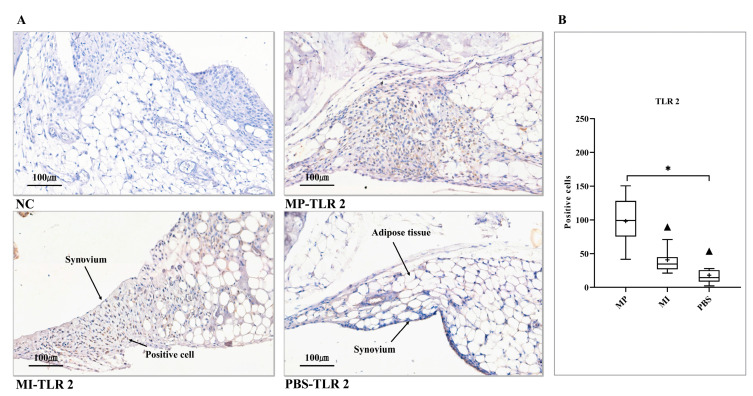
Expression of TLR 2 in the synovial layer of knee joints. (**A**) NC, no positive cell was found. MP-TLR 2 showed thickened synovium and numerous positive cells. MI-TLR 2, positive cell infiltration was observed in the synovium. PBS-TLR 2, scattered positive cells were found in the synovial membrane. (**B**) Histomorphometrical analysis of TLR 2 expression levels in three groups (Kruskal–Wallis test (Dunn’s test)). (+) and (▲) symbols represent the mean and outlier values, respectively. NC, no primary antibody control; MP, metal particles; MI, metal ions; PBS, phosphate-buffered saline; TLR, Toll-like receptor. (Scale bars = 100 µm; * = *p* < 0.0167).

Thickened synovial tissues were found in both the MP and the MI groups. Several TLR 2-positive cells were also visualized in both the MP and the MI groups (Figure 2A). However, from the results of the histomorphometrical analysis, only the MP group showed significantly upregulated TLR 2 expression levels in comparison with the PBS group (*p* < 0.0167) (Figure 2B).

### 3.3. Expression of TLR 4

**Figure 3:**
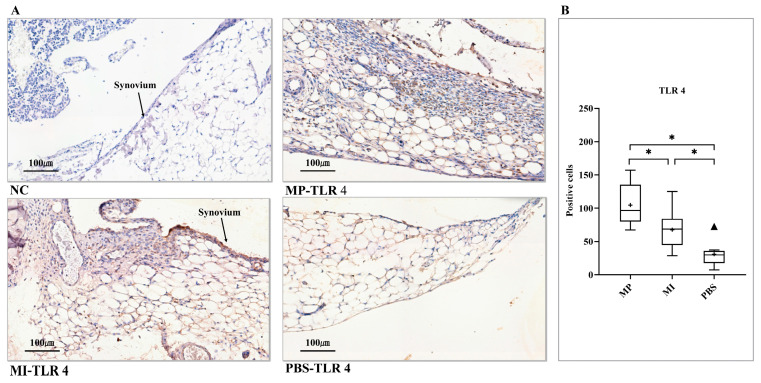
Expression of TLR 4 in the synovial layer of knee joints. (**A**) NC, no positive cell was found. MP-TLR 4 showed inflammatory cell infiltration accompanied by the adjacent adipose tissue loss. MI-TLR 4, some positive cells were observed in the thickened synovium. PBS-TLR 4, scattered positive cells were found in the synovium. (**B**) Histomorphometrical analysis of TLR 4 expression levels in three groups (One-way ANOVA (Tukey’s test)). (+) and (▲) symbols represent the mean and outlier values, respectively. NC, no primary antibody control; MP, metal particles; MI, metal ions; PBS, phosphate-buffered saline; TLR, Toll-like receptor. (Scale bars = 100 µm; * = *p* < 0.0167).

Synovial hyperplasia conjunction with apparent inflammatory cell infiltration into the adjacent adipose tissue, was present in the MP group. Most of the cellular infiltrates contained abundant monocyte/macrophage-like cells and some fibroblast-like cells; a considerable number of them were positive for TLR 4 staining. Regarding the MI group, a large number of positive cells were also found in a thickened synovium; however, for the PBS group, only scattered positive cells were found in the synovium (Figure 3A). According to the histomorphometrical analysis of TLR 4, expression levels in the MP group were considerably elevated in comparison with the MI group and the PBS group. Meanwhile, the MI group had a higher TLR 4 expression compared with the PBS group (*p* < 0.0167) (Figure 3B).

### 3.4. Expression of TLR 5

**Figure 4:**
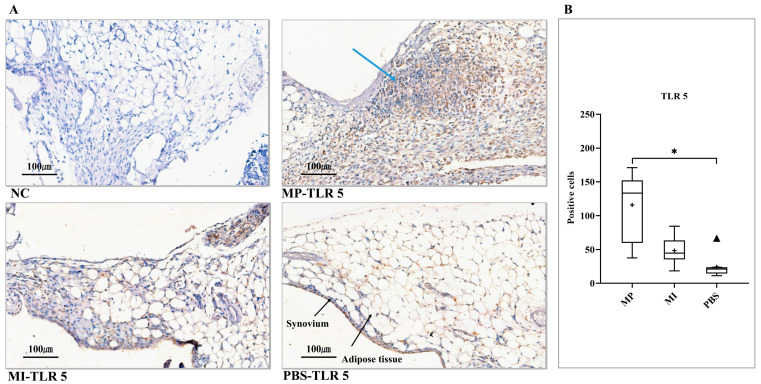
Expression of TLR 5 in the synovial layer of knee joints. (**A**) NC, no positive cell was found. MP-TLR 5, newly formed granulomatous tissue (blue arrow). MI-TLR 5, some positive cells were observed in the synovium. PBS-TLR 5, scattered positive cells were found in the synovial membrane. (**B**) Histomorphometrical analysis of TLR 5 expression levels in three groups (Kruskal–Wallis test (Dunn’s test)). (+) and (▲) symbols represent the mean and outlier values, respectively. NC, no primary antibody control; MP, metal particles; MI, metal ions; PBS, phosphate-buffered saline; TLR, Toll-like receptor. (Scale bars = 100 µm; * = *p* < 0.0167).

Due to the exposure to metal particles, non-specific granulation tissue was seen, quite similar to granulomatous tissues (pseudotumors) obtained from revision surgeries [38,39]. Additionally, accompanied by the formation of granulomatous structures, extensive adjacent adipose tissue seemed to disappear in the MP group (Figure 4A, MP-TLR 5). Although there was no granulation tissue in terms of the MI group, a thickened synovial layer was observed (Figure 4A, MI-TLR 5). For the histomorphometrical analysis, only the MP group had significantly elevated expression of TLR 5 compared to the PBS group (*p* < 0.0167) (Figure 4B).

### 3.5. Expression of TLR 6

**Figure 5:**
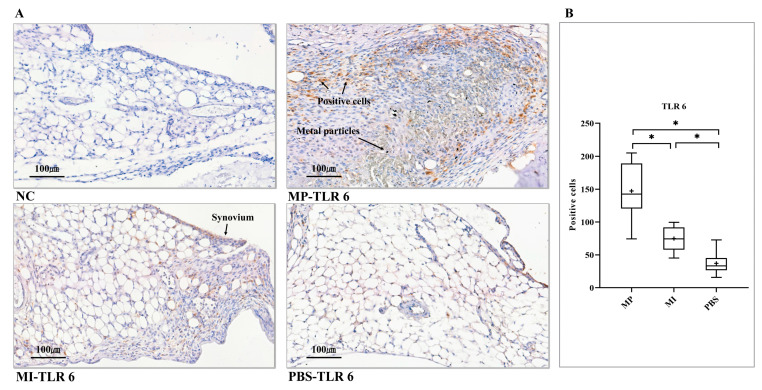
Expression of TLR 6 in the synovial layer of knee joints. (**A**) NC, no positive cell was found. MP-TLR 6, debris-induced necrotic tissue contained greenish corrosion particles and black metal particles. MI-TLR 6, some positive cells were observed in the thickened synovium. PBS-TLR 6, scattered positive cells were found in the synovial membrane. (**B**) Histomorphometrical analysis of TLR 6 expression levels in three groups (One-way ANOVA (Tukey’s test)). (+) symbol represents the mean value. NC, no primary antibody control; MP, metal particles; MI, metal ions; PBS, phosphate-buffered saline; TLR, Toll-like receptor. (Scale bars = 100 µm; * = *p* < 0.0167).

The debris-induced necrotic tissue was present in the MP group, characterized by a central area of necrosis containing greenish corrosion metal particles, black metal particles, and numerous debris-loaded macrophages (Figure 5A, MP-TLR 6). Commonly, corrosion metal particles appear greenish, while conventional metal particles were mainly black at microscopy [40], consistent with what we found in the necrotic tissue of this study. Around the necrotic tissue, numerous TLR 6-positive macrophage-like cells and TLR 6-positive spindle-shaped, fibroblast-like cells were observed. In the MI group, many TLR 6-positive cells were also found in the obvious thickened synovium (Figure 5A, MI-TLR 6). In terms of the histomorphometrical analysis, the MP group showed greatly increased TLR 6-positive cells compared to the MI group and the control group (PBS), and meanwhile, the MI group had more TLR 6-positive cells compared to the control group (*p* < 0.0167) (Figure 5B).

## 4. Discussion

The histopathological findings partially refuted the initial hypothesis for this investigation. The MP group, rather than the MI group, showed dramatically elevated expression levels of all TLRs used in this study compared with the PBS group (control). Even higher expression rates of TLRs 1, 4, and 6 were observed in the MP group compared to the MI group. Only upregulated expression levels of TLRs 4 and 6 were found for the MI group compared to the PBS group.

The identification of inflammatory characteristics in the synovial-like interface membrane via standardized histopathological analysis plays an imperative role in understanding the biological reactions contributing to aseptic loosening in total joint replacement [40,41]. Furthermore, thanks to histopathological techniques, the quantification of particular cell types in the periprosthetic synovial-like tissue has been used to establish possible patterns/thresholds for specific pathological reactions, e.g., low-grade bacterial infections diagnosis by quantifying CD15-positive cells [41]. However, periprosthetic tissue samples occasionally obtained from revision surgeries are scarce, which is a currently substantial obstacle to further understanding periprosthetic biological reactions [42]. In light of the situation, a murine model was established in our institution that could closely mimic periprosthetic biological reactions upon stimulation with wear particles [9,43,44]. Especially in this study, greenish corrosion metallic particles, black metallic particles, inflammatory cell infiltration, debris-induced necrotic tissue, and even the granulation tissue (pseudotumor-like tissues), consistent with the clinical scenario [40,42,45,46], were found in the synovial layer of the murine knee joints. This seems to reflect the superiority of the used inflammatory in vivo model. Additionally, compared with numerous in vitro cell culture studies involving sterile inflammation to wear debris, the animal model not only can reflect complicated cellular and tissue interactions but can also resemble the dynamic process of joints. With regard to metallic materials, because TLRs are usually activated by PAMPs [15], endotoxin-free Co28Cr6Mo materials were used to eliminate interfering factors in this study. When Zysk et al. [47] first applied this murine model to observe inflammatory response caused by wear debris, they initially evaluated the severity of inflammation after polystyrene particle injection at different incubation time points (days 1, 2, 3, 5, 7, 21, and 63). Based on their main results of synovial microcirculation using intravital microscopy, the inflammatory response was strongest after seven days of the intra-articular injection. Due to its high sensitivity, we choose the incubation time of seven days directly in the present study.

Expression levels of TLR 4 and TLR 6 were significantly elevated after metallic ion stimulation compared to the control group in our murine model. Similarly, upon stimulation of nickel (Ni), enhanced expression levels of TLR 4 in mice were also observed by another study [48]. Moreover, Samelko et al. [32] once observed an intense TLR 4-based inflammation after a Co28Cr6Mo/LPS+ or Co28Cr6Mo metal challenge in an established murine calvaria model. Nevertheless, one research involving contact allergy has indicated that Co^2+^ or Ni^2+^ ions (concentration, 1.5 mM), such as PAMPs, trigger an inflammatory response by directly activating human TLR 4 but not murine TLR 4 [49]. Because the study showed that, unlike TLR 4 in humans, the TLR 4 of mice lacks the non-conserved histidines 456 and 458, which are required for direct activation by Ni^2+^ and Co^2+^ ions [50]. Briefly, direct TLR 4 activation by Co^2+^ ions or Ni^2+^ ions was species-specific. Therefore, the enhanced TLR 4 expression levels in our murine model seem not to be due to the direct effect of Co^2+^ and Ni^2+^ ions. Considering that Co28Cr6Mo alloys used in this study also contain other elements, such as chromium and molybdenum, the increase of TLR 4 is probably directly triggered by these elements. However, due to the complicated physical and chemical properties, the biological effects of metals seem not to be limited to a single manner. In addition to the direct ligand-receptor interaction, metallic ions at high concentrations can also indirectly activate TLRs, which is mainly achieved by triggering the release of endogenous ligands of TLRs [11], such as some DAMPs released from damaged or dying cells [51]. Similarly, the concentration of metallic ions in this study is based on patients scheduled for a revision arthroplasty [37], which might be inadequate for murine knee joints, probably then resulting in the release of DAMPs subsequent activation of TLR 4 and TLR 6. Clinically, the metallic ion levels typically seen in patients with well-functioning implants are not close to toxic levels [52]. However, there are higher concentrations of metallic ions in the joint fluid of patients who need revision surgery, which is generally toxic [37,53]. Moreover, expression levels of TLR 4 and TLR 6 are upregulated in periprosthetic tissues obtained from these revision surgeries [19,54]. As outlined above, for TLR activation, in addition to the direct ligand-receptor interaction, the effect of metallic ion concentration should also not be underestimated clinically or experimentally.

The authors also observed that the MP group had more TLR 4- and 6-positive cells than the MI group. In this study, greenish corrosion particles in the necrotic tissue area suggested that metallic particles could be a metallic ion reservoir. A certain level of metallic ions was continuously released from metallic particles within the synovial tissue or cells via electrochemical corrosion. However, the initially high level of metallic ions in the MI group might be inevitably disseminated via blood vessels and lymphatics and gradually quenched throughout the host body [5]. Continuously released metal ions in the MP group seem to provide a reasonable explanation for the higher expression levels of TLR 4 and TLR 6 in the MP group than that in the MI group. Commonly, during manufacturing, the Co28Cr6Mo implants will form a protective oxide layer (1–4 nm thick), mainly including CoO and Cr_2_O_3,_ to prevent severe corrosion [33]. After the implantation, the oxide layer can be gradually destroyed because of the wear and mechanical loading, exposing the un-oxidized metal to the physiological environment. Anyway, the corrosion process of Co28Cr6Mo implants is relatively slow because of the oxide film [55]. In the present study, the authors use Co28Cr6Mo nanoparticles to conduct intra-articular injection directly. Unlike Co28Cr6Mo implants, these nanoparticles could be phagocytized by macrophages and be exposed to reactive oxygen species within cells. Reactive oxygen species within macrophages, such as superoxide and hypochlorous acid, underwent redox reactions with nanoparticles [56]. During this process, cells were damaged, and a certain level of ions might be rapidly released from metallic particles. The necrotic tissue and greenish corrosion particles were observed in the MP group only seven days after injection; this finding might be related to the redox reaction of metal particles described above.

In addition to the corrosion process, the effects of some distinguishing physical characteristics of metal particles relative to the MI group and the PBS group also should not be neglected. Numerous physical properties of wear particles, including the size, shape, dose, and volume, can influence biological reactions around the prosthesis [57,58,59]. Quantities of studies have shown that nanoparticles can damage cell membranes (thickness, 4–10 nm) by perforating them [60]. Even some studies showed the damaged holes caused by nanoparticles on the cell membrane using hopping probe ion conductance microscopy [61]. As described above, metal nanoparticles (61.25 ± 18.47 nm) used in this study probably damage cellular membranes directly under some particular situation, especially when murine joints are under tremendous pressure (in strong motion). After damage, cells activate various TLRs of adjacent immune cells and recruit more immunocompetent cells, e.g., macrophages, by releasing DAMPs and inflammatory mediators [62]. In addition to phagocytizing nanoparticles, recruited macrophages express various TLRs according to surrounding danger signals [59,62]. In the present study, the MP group showed more TLR 1-positive cells than the MI and control groups; however, no significant difference was found between the MI and control groups. Therefore, the higher levels of TLR 1 in the MP group might be attributable to unique characteristics of metallic particles that differ from metallic ions and PBS, but more comprehensive studies are needed for further elucidation.

Because three cytokines (IL-6, IL-1β, and TNF-α) are critical pro-inflammatory mediators present in periprosthetic tissues and are even relevant to subsequent aseptic implant loosening, our research group once used them as pro-inflammatory markers to assess the extent of inflammatory response in the MP, MI and PBS groups [34]. The previous results showed that expression levels of IL-6, IL-1β, and TNF-α in the MP group were upregulated significantly compared to the control group, which was consistent with the results of TLR 2 and TLR 5 in this investigation. Greenfield et al. [63] showed that TLR 2^−/−^ macrophages of mice secreted fewer TNF-α than normal macrophages after titanium particle exposure in vitro. Additionally, Kassem et al. [64] indicated that TLR 5 is a potential key mediator in the process of inflammation-induced osteoclastogenesis and osteolysis. These data strongly support the critical roles of TLR 2 and TLR 5 in aseptic inflammation caused by metallic particles.

Our institution also observed enhanced expression of TLR 2 in the synovial membrane upon the stimulation of ultra-high molecular weight polyethylene (UHMWPE) wear particles, which was consistent with our results in the MP group of this study [20]. However, no increased TLR 1 and TLR 4 were observed in the previous study. One possible explanation for the difference between studies is that, unlike Co28Cr6Mo particles, UHMWPE particles do not release ions due to an electrochemical corrosion process. Furthermore, different size and shape parameters of Co28Cr6Mo particles and UHMWPE particles used in these experiments are also potential factors that can intensely influence biological reactions in the synovial layer of mice. Finally, both rounded macrophage-like cells and spindle-shaped fibroblast-like cells with positive reactions were counted in this study. However, the investigation concerning UHMWPE particles only focused on round macrophage-like cells.

In summary, the corrosion particles, dense inflammatory infiltration, and increased TLR-positive cells observed in the MP group suggest that the physical and chemical properties of wear debris may play a critical role in periprosthetic biological reactions. Briefly, wear debris seems to be the culprit. Based on this, in terms of the prosthetic design, preventing the release of particles from implants would be an extremely effective strategy to prolong the longevity of prostheses. In addition, the development of anti-inflammatory strategies in periprosthetic tissues may also be helpful. Because numerous TLR-positive cells were found after metallic particle stimulation in this investigation, specific pharmacologic blocking of single TLR or multiple TLRs may be effective for mitigating wear debris-induced inflammation. In future studies, after a specific pharmacological block, reassessing the severity of inflammation in this murine model may clarify the potential feasibility.

Some limitations exist in the present study that needs to be considered. Although this murine model can highly resemble inflammatory reactions in the synovial-like interface membrane around an endo-prosthetic implant, involving the subsequent osteolysis, the murine model cannot allow for direct conclusions. Moreover, referring to the national animal laws, a concentration gradient-related analysis that would require a particularly high number of test animals was not carried out in this study. The concentration of metallic particles and ions used in this study is based on previous experiments and clinical studies [37,65]. The effects of different metallic particles and metallic ion concentrations on sterile inflammation will be investigated in future studies. Although we provide some new insights to clarify the effects of metallic particles and metallic ions on TLR expression in the present study, the exact patterns of TLR activation related to metallic byproducts (e.g., direct or indirect stimulation, the difference between single metallic elements) still need to be further elucidated, which are also the point of our further research.

## 5. Conclusions

The results obtained in this investigation suggest that especially metallic wear particles result in a severe inflammatory response and high expression levels of surface TLRs. Additionally, greenish corrosion particles found in the necrotic tissue indicate that metallic particles might release a certain level of locally toxic ions in the physiological environment of the synovial layer. Significantly higher levels of TLRs 4 and 6 were observed after the metallic ions were injected into the murine knee joints. The present results reveal apparent differences in TLR expression between metallic particles and ions in vivo.

## Figures and Tables

**Table 1:** Metal ion concentrations in the stock solution.

Content In	Co	Cr	Mo	Ni
stock solution	13.7 mg/L	4.3 mg/L	0.8 mg/L	1.7 mg/L

Total ion concentrations of the Co28Cr6Mo stock solution were determined as 20.5 mg/L.

## Data Availability

The data presented in this study are available on request from the corresponding author.

## Supplementary Materials

The following are available online at https://www.mdpi.com/article/10.3390/jcm10163489/s1, Figure S1: Results of the positive control staining.

## References

[1] Jonitz-Heincke A., Tillmann J., Klinder A., Krueger S., Kretzer J.P., Høl P.J., Paulus A.C., Bader R. (2017). The Impact of Metal Ion Exposure on the Cellular Behavior of Human Osteoblasts and PBMCs: In Vitro Analyses of Osteolytic Processes. Materials.

[2] Goodman S.B., Gallo J., Gibon E.F., Takagi M. (2019). Diagnosis and management of implant debris-associated inflammation. Expert Rev. Med. Devices.

[3] Price A.J., Alvand A., Troelsen A., Katz J.N., Hooper G., Gray A., Carr A., Beard D. (2018). Knee replacement. Lancet.

[4] Learmonth I.D., Young C., Rorabeck C. (2007). The operation of the century: Total hip replacement. Lancet.

[5] Sansone V. (2013). The effects on bone cells of metal ions released from orthopaedic implants. A review. Clin. Cases Miner. Bone Metab..

[6] Krenn V., Morawietz L., Perino G., Kienapfel H., Ascherl R., Hassenpflug G., Thomsen M., Thomas P., Huber M., Kendoff D. (2014). Revised histopathological consensus classification of joint implant related pathology. Pathol. Res. Pract..

[7] Mahendra G., Pandit H., Kliskey K., Murray D., Gill H., Athanasou N. (2009). Necrotic and inflammatory changes in metal-on-metal resurfacing hip arthroplasties. Acta Orthop..

[8] Goodman S.B., Gibon E., Pajarinen J., Lin T., Keeney M., Ren P.-G., Nich C., Yao Z., Egashira K., Yang F. (2014). Novel biological strategies for treatment of wear particle-induced periprosthetic osteolysis of orthopaedic implants for joint replacement. J. R. Soc. Interface.

[9] Utzschneider S., Lorber V., Dedic M., Paulus A.C., Schröder C., Gottschalk O., Schmitt-Sody M., Jansson V. (2014). Biological activity and migration of wear particles in the knee joint: An in vivo comparison of six different polyethylene materials. J. Mater. Sci. Mater. Electron..

[10] Gibon E., Goodman S.B. (2016). The Biologic Response to Bearing Materials. Orthop. Knowl. Online J..

[11] Takagi M., Takakubo Y., Pajarinen J., Naganuma Y., Oki H., Maruyama M., Goodman S.B. (2017). Danger of frustrated sensors: Role of Toll-like receptors and NOD-like receptors in aseptic and septic inflammations around total hip replacements. J. Orthop. Transl..

[12] Keegan G.M., Learmonth I.D., Case C.P. (2007). Orthopaedic metals and their potential toxicity in the arthroplasty patient: A review of current knowledge and future strategies. J. Bone Jt. Surgery. Br. Vol..

[13] Granchi D., Savarino L.M., Ciapetti G., Baldini N. (2017). Biological effects of metal degradation in hip arthroplasties. Crit. Rev. Toxicol..

[14] Cobelli N., Scharf B., Crisi G.M., Hardin J., Santambrogio L. (2011). Mediators of the inflammatory response to joint replacement devices. Nat. Rev. Rheumatol..

[15] Kawai T., Akira S. (2010). The role of pattern-recognition receptors in innate immunity: Update on Toll-like receptors. Nat. Immunol..

[16] Lähdeoja T., Pajarinen J., Kouri V.-P., Sillat T., Salo J., Konttinen Y.T. (2009). Toll-like receptors and aseptic loosening of hip endoprosthesis-a potential to respond against danger signals?. J. Orthop. Res..

[17] Akira S., Takeda K., Kaisho T. (2001). Toll-like receptors: Critical proteins linking innate and acquired immunity. Nat. Immunol..

[18] Amer L.D., Saleh L.S., Walker C., Thomas S., Janssen W.J., Alper S., Bryant S.J. (2019). Inflammation via myeloid differentiation primary response gene 88 signaling mediates the fibrotic response to implantable synthetic poly(ethylene glycol) hydrogels. Acta Biomater..

[19] Takagi M., Tamaki Y., Hasegawa H., Takakubo Y., Konttinen L., Tiainen V.-M., Lappalainen R., Konttinen Y.T., Salo J. (2007). Toll-like receptors in the interface membrane around loosening total hip replacement implants. J. Biomed. Mater. Res. Part A.

[20] Paulus A.C., Frenzel J., Ficklscherer A., Roßbach B.P., Melcher C., Jansson V., Utzschneider S. (2013). Polyethylene wear particles induce TLR 2 upregulation in the synovial layer of mice. J. Mater. Sci. Mater. Med..

[21] Grandjean-Laquerriere A., Tabary O., Jacquot J., Richard D., Frayssinet P., Guenounou M., Laurent-Maquin D., Laquerriere P., Gangloff S. (2007). Involvement of toll-like receptor 4 in the inflammatory reaction induced by hydroxyapatite particles. Biomaterials.

[22] Pajarinen J., Mackiewicz Z., Pöllänen R., Takagi M., Epstein N.J., Ma T., Goodman S.B., Konttinen Y.T. (2009). Titanium particles modulate expression of Toll-like receptor proteins. J. Biomed. Mater. Res. Part A.

[23] Valladares R.D., Nich C., Zwingenberger S., Li C., Swank K.R., Gibon E., Rao A.J., Yao Z., Goodman S.B. (2013). Toll-like receptors-2 and 4 are overexpressed in an experimental model of particle-induced osteolysis. J. Biomed. Mater. Res. Part A.

[24] Kawai T., Akira S. (2006). TLR signaling. Cell Death Differ..

[25] Vijay K. (2018). Toll-like receptors in immunity and inflammatory diseases: Past, present, and future. Int. Immunopharmacol..

[26] Bonham K., Orzalli M.H., Hayashi K., Wolf A.I., Glanemann C., Weninger W., Iwasaki A., Knipe D.M., Kagan J.C. (2014). A Promiscuous Lipid-Binding Protein Diversifies the Subcellular Sites of Toll-like Receptor Signal Transduction. Cell.

[27] Triantafilou M., Gamper F.G., Haston R.M., Mouratis M.A., Morath S., Hartung T., Triantafilou K. (2006). Membrane Sorting of Toll-like Receptor (TLR)-2/6 and TLR2/1 Heterodimers at the Cell Surface Determines Heterotypic Associations with CD36 and Intracellular Targeting. J. Biol. Chem..

[28] Molteni M., Gemma S., Rossetti C. (2016). The Role of Toll-Like Receptor 4 in Infectious and Noninfectious Inflammation. Mediat. Inflamm..

[29] Merola M., Affatato S. (2019). Materials for Hip Prostheses: A Review of Wear and Loading Considerations. Materials.

[30] Catelas I., Petit A., Vali H., Fragiskatos C., Meilleur R., Zukor D.J., Antoniou J., Huk O.L. (2005). Quantitative analysis of macrophage apoptosis vs. necrosis induced by cobalt and chromium ions in vitro. Biomaterials.

[31] Paulus A.C., Ebinger K., Cheng X., Haßelt S., Weber P., Kretzer J.P., Bader R., Utzschneider S. (2019). Local Biological Reactions and Pseudotumor-Like Tissue Formation in relation to Metal Wear in a Murine In Vivo Model. BioMed Res. Int..

[32] Samelko L., Landgraeber S., McAllister K., Jacobs J., Hallab N.J. (2017). TLR4 (not TLR2) dominate cognate TLR activity associated with CoCrMo implant particles. J. Orthop. Res..

[33] Eliaz N. (2019). Corrosion of Metallic Biomaterials: A Review. Materials.

[34] Cheng X., Dirmeier S.C., Haßelt S., Baur-Melnyk A., Kretzer J.P., Bader R., Utzschneider S., Paulus A.C. (2020). Biological Reactions to Metal Particles and Ions in the Synovial Layer of Mice. Materials.

[35] Athanasou N.A. (2016). The pathobiology and pathology of aseptic implant failure. Bone Jt. Res..

[36] Catelas I., Wimmer M.A., Utzschneider S. (2011). Polyethylene and metal wear particles: Characteristics and biological effects. Semin. Immunopathol..

[37] De Smet K., De Haan R., Calistri A., Campbell P., Ebramzadeh E., Pattyn C., Gill H. (2008). Metal Ion Measurement as a Diagnostic Tool to Identify Problems with Metal-on-Metal Hip Resurfacing. J. Bone Jt. Surg. Am. Vol..

[38] Orr C., Vieira-Sousa E., Boyle D.L., Buch M., Buckley C.D., Cañete J.D., Catrina A.I., Choy E.H.S., Emery P., Fearon U. (2017). Synovial tissue research: A state-of-the-art review. Nat. Rev. Rheumatol..

[39] Goodman S.B., Gallo J. (2019). Periprosthetic Osteolysis: Mechanisms, Prevention and Treatment. J. Clin. Med..

[40] Perino G., Sunitsch S., Huber M., Ramirez D., Gallo J., Vaculova J., Natu S., Kretzer J.P., Müller S., Thomas P. (2018). Diagnostic guidelines for the histological particle algorithm in the periprosthetic neo-synovial tissue. BMC Clin. Pathol..

[41] Krenn V., Perino G., Wienert S., Saberi D., Hügle T., Hopf F., Huber M., Krenn V. (2016). Histopathologische Diagnostik von Gelenkendoprothesen-assoziierten Erkrankungen. Der Hautarzt.

[42] Gallo J., Vaculova J., Goodman S.B., Konttinen Y.T., Thyssen J.P. (2014). Contributions of human tissue analysis to understanding the mechanisms of loosening and osteolysis in total hip replacement. Acta Biomater..

[43] Utzschneider S., Becker F., Grupp T.M., Sievers B., Paulus A., Gottschalk O., Jansson V. (2010). Inflammatory response against different carbon fiber-reinforced PEEK wear particles compared with UHMWPE in vivo. Acta Biomater..

[44] Lorber V., Paulus A.C., Buschmann A., Schmitt B., Grupp T.M., Jansson V., Utzschneider S. (2013). Elevated cytokine expression of different PEEK wear particles compared to UHMWPE in vivo. J. Mater. Sci. Mater. Electron..

[45] Burkandt A., Katzer A., Thaler K., Von Baehr V., Friedrich R.E., Rüther W., Amling M., Zustin J. (2011). Proliferation of the synovial lining cell layer in suggested metal hypersensitivity. In Vivo.

[46] Gallo J., Goodman S., Konttinen Y., Wimmer M., Holinka M. (2013). Osteolysis around total knee arthroplasty: A review of pathogenetic mechanisms. Acta Biomater..

[47] Zysk S.P., Gebhard H.H., Pellengahr C., Refior H.J., Plitz W., Messmer K., Veihelmann A. (2003). Inflammatory responses to wear particles in vivo: A novel model in the murine knee joint. Der Orthop..

[48] Bannon D.I., Bao W., Turner S.D., McCain W.C., Dennis W.E., Wolfinger R., Perkins E.E., Abounader R. (2020). Gene expression in mouse muscle over time after nickel pellet implantation. Metallomics.

[49] Raghavan B., Martin S.F., Esser P., Goebeler M., Schmidt M. (2012). Metal allergens nickel and cobalt facilitate TLR4 homodimerization independently of MD2. EMBO Rep..

[50] Schmidt M., Raghavan B., Müller V., Vogl T., Fejer G., Tchaptchet S., Keck S., Kalis C., Nielsen P.J., Galanos C. (2010). Crucial role for human Toll-like receptor 4 in the development of contact allergy to nickel. Nat. Immunol..

[51] Herrero-Beaumont G., Pérez-Baos S., Pernaute O.S., Roman-Blas J.A., Lamuedra A., Largo R. (2019). Targeting chronic innate inflammatory pathways, the main road to prevention of osteoarthritis progression. Biochem. Pharmacol..

[52] Back D.L., Young D.A., Shimmin A.J. (2005). How Do Serum Cobalt and Chromium Levels Change after Metal-on-Metal Hip Resurfacing?. Clin. Orthop. Relat. Res..

[53] Jacobs J.J., Skipor A.K., Patterson L.M., Hallab N.J., Paprosky W.G., Black J., Galante J.O. (1998). Metal Release in Patients Who Have Had a Primary Total Hip Arthroplasty. A Prospective, Controlled, Longitudinal Study. J. Bone Jt. Surg. Am. Vol..

[54] Li D., Wang H., Li Z., Wang C., Xiao F., Gao Y., Zhang X., Wang P., Peng J., Cai G. (2018). The inhibition of RANKL expression in fibroblasts attenuate CoCr particles induced aseptic prosthesis loosening via the MyD88-independent TLR signaling pathway. Biochem. Biophys. Res. Commun..

[55] Liao Y., Hoffman E., Wimmer M., Fischer A., Jacobs J., Marks L. (2012). CoCrMo metal-on-metal hip replacements. Phys. Chem. Chem. Phys..

[56] Dayem A.A., Hossain M.K., Bin Lee S., Kim K., Saha S.K., Yang G.-M., Choi H.Y., Cho S.-G. (2017). The Role of Reactive Oxygen Species (ROS) in the Biological Activities of Metallic Nanoparticles. Int. J. Mol. Sci..

[57] Schröder C., Reinders J., Zietz C., Utzschneider S., Bader R., Kretzer J.P. (2013). Characterization of polyethylene wear particle: The impact of methodology. Acta Biomater..

[58] Utzschneider S., Paulus A., Datz J.-C., Schroeder C., Sievers B., Wegener B., Jansson V. (2009). Influence of design and bearing material on polyethylene wear particle generation in total knee replacement. Acta Biomater..

[59] Paulus A.C., Haßelt S., Jansson V., Giurea A., Neuhaus H., Grupp T.M., Utzschneider S. (2016). Histopathological Analysis of PEEK Wear Particle Effects on the Synovial Tissue of Patients. BioMed Res. Int..

[60] Shukhnova A., Bozrova S., Sokolov P., Berestovoy M., Karaulov A., Nabiev I. (2018). Dependence of Nanoparticle Toxicity on Their Physical and Chemical Properties. Nanoscale Res. Lett..

[61] Ruenraroengsak P., Novak P., Berhanu D., Thorley A.J., Valsami-Jones E., Gorelik J., Korchev Y., Tetley T.D. (2011). Respiratory epithelial cytotoxicity and membrane damage (holes) caused by amine-modified nanoparticles. Nanotoxicology.

[62] Zhang X., Mosser D.M. (2007). Macrophage activation by endogenous danger signals. J. Pathol..

[63] Greenfield E.M., Beidelschies M.A., Tatro J.M., Goldberg V.M., Hise A. (2010). Bacterial Pathogen-associated Molecular Patterns Stimulate Biological Activity of Orthopaedic Wear Particles by Activating Cognate Toll-like Receptors. J. Biol. Chem..

[64] Kassem A., Henning P., Kindlund B., Lindholm C., Lerner U.H. (2015). TLR5, a novel mediator of innate immunity-induced osteoclastogenesis and bone loss. FASEB J..

[65] Zysk S.P., Gebhard H.H., Kalteis T., Schmitt-Sody M., Jansson V., Messmer K., Veihelmann A. (2005). Particles of All Sizes Provoke Inflammatory Responses In Vivo. Clin. Orthop. Relat. Res..

