# Supplementary material for: The Expression Levels of Toll-like Receptors after Metallic Particle and Ion Exposition in the Synovium of a Murine Model
Source: J Clin Med. 2021 Aug 7;10(16):3489. doi: 10.3390/jcm10163489 (PMC8396889; doi:10.3390/jcm10163489)
Supplement: Supplementary file 1 [file jcm-10-03489-s001.zip › jcm-1263275-supplementary.pdf]

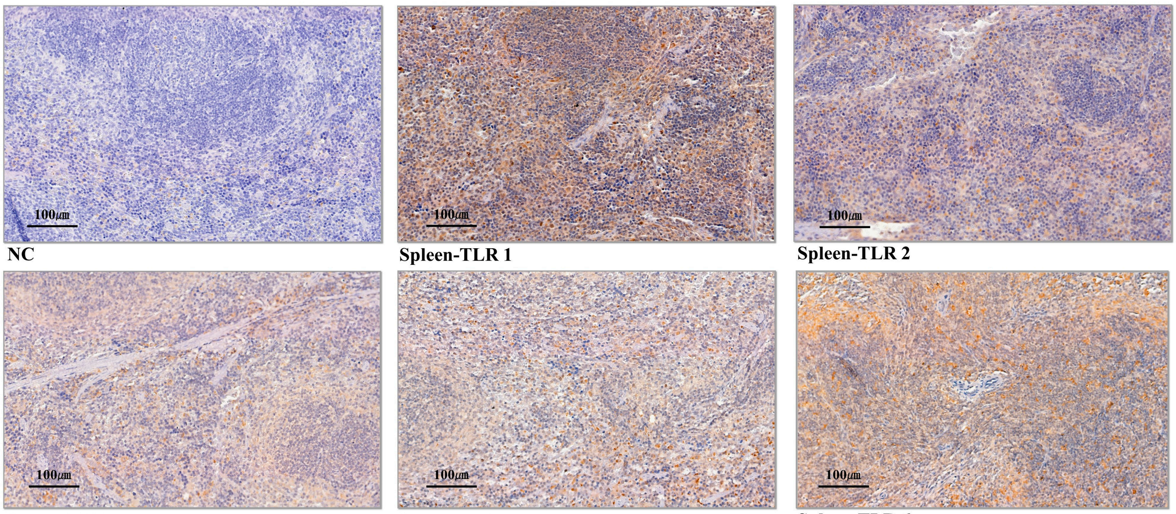

NC
Spleen-TLR 1
Spleen-TLR 2
Spleen-TLR 4
Spleen-TLR 5
Spleen-TLR 6

Figure S1. Results of the positive control staining. The spleen, an immune organ, expresses all antigens that were our focus in this study. Therefore, splenic tissue was used as a positive control. In the pilot experiment, the splenic sample was used to check if the staining assay was working correctly. In the subsequent formal experiments, one splenic sample was stained again in each staining batch to avoid false-negative results. NC, no primary antibody control, only no primary antibody was added, which could exclude some false-positive results. (Scale bars = 100 μm).
